# The Influence of Menstrual Cycle Phases on Postconcussion Outcomes and Symptom Reporting: A Scoping Review

**DOI:** 10.1111/sms.70093

**Published:** 2025-06-25

**Authors:** Gina Carr, Marie‐Therese Fleddermann

**Affiliations:** ^1^ Department of Movement Science and Training in Sports, Institute of Sport Sciences Goethe University Frankfurt Germany

**Keywords:** diagnostic, estrogen, hormones, mTBI, progesterone, woman

## Abstract

Concussion is common in sports and often shows sex‐based differences in symptom severity and recovery, with female athletes experiencing more severe, prolonged symptoms. Hormonal changes during the menstrual cycle may play a role in concussion, but research on this topic has been limited. Therefore, this scoping review aimed to investigate whether the menstrual cycle phase—both at the time of injury and after injury—influences postconcussion outcomes and symptom reporting, and whether menstrual cycle phases influence baseline assessments in the absence of injury. This review was conducted according to PRISMA‐ScR guidelines and included studies on the influence of menstrual cycle phases on concussion‐related outcomes retrieved from PubMed, Web of Science, and BISp‐Surf databases. Five studies involving 774 females identified variations in symptom severity and health outcomes across menstrual cycle phases, with differences between the luteal and follicular phases. Findings suggest that hormonal fluctuations, particularly the withdrawal of elevated progesterone during the luteal phase, may contribute to worse postconcussion symptoms and outcomes, with these hormone levels at the time of injury emerging as a potential predictor of recovery severity. Baseline assessment evaluations should account for menstrual cycle phase, because this influences symptom variability and severity. This review underscores the importance of incorporating menstrual cycle phases into concussion management strategies in order to enhance diagnostic accuracy and recovery approaches for female athletes. Addressing these hormonal influences can advance both research and practice in the management of concussion.

## 1 Introduction

A concussion represents a subset of mild traumatic brain injury (mTBI) and is typically at the milder end of the brain injury spectrum [1, 2, 3]. It is common in sports, and an estimated 3.8 million concussions occur annually in the United States [4] and approximately 44 000 in Germany [5], and around 40 000 in Australia [6]—most commonly in football, hockey, rugby, soccer, and basketball [4]. It is defined as a traumatically induced, temporary disturbance of brain function that involves a complex pathophysiological process characterized by a range of symptoms including headache, dizziness, nausea, blurred vision, and cognitive issues such as memory and attention deficits [1, 2, 3, 4]. Concussions frequently result from a direct impact to the neck, face, head or other parts of the body with an impulsive force transmitted to the brain. Although concussion can cause neuropathological changes, these are generally classified as functional disturbances (e.g., headache, dizziness) rather than structural damage. As a result, they are often undetectable through standard diagnostic imaging [1, 2, 4] and despite increased public awareness, up to 50% of concussions remain unreported [4], underscoring the need for further research into their nature, treatment, and management. The current literature shows that sex in particular plays a role in concussion incidence, risk and recovery [7]. In addition to an increased risk of female athletes sustaining a concussion during games compared to their male counterparts [8], it has been shown that females also tend to experience more severe concussion‐related symptoms [7, 9, 10, 11, 12] and require longer recovery periods [13, 14]. For example, Broshek et al. [9] found that females reported significantly more symptoms following a concussion compared to males, including issues such as concentration problems, fatigue, lightheadedness, and seeing flyspecks; and they were cognitively impaired approximately 1.5 times more often than males. Although the existence of sex‐based differences in postconcussion outcomes has been known for some time, the underlying mechanisms remain poorly understood [15]. Whereas research has begun to acknowledge the distinctive physiological and psychosocial responses of female athletes to concussion [16], further investigation is required to fully elucidate these differences. One factor that may contribute to these differences is the potential influence of ovarian sex hormones on postconcussion outcomes [7, 9, 10]. Females, particularly during their reproductive years when ovarian sex hormones exhibit greater fluctuations, report more symptoms after sustaining a concussion and may experience inferior recovery outcomes due to hormonal fluctuations, particularly those related to the menstrual cycle (MC) [10, 13]. Therefore, hormonal influences associated with the MC offer further insight and may potentially influence not only the *severity of reported symptoms following a concussion* but also the *degree or expression of the symptom level at the time of the injury*.

The typical ovulatory cycle has a variability between 21 and 35 days [17, 18], and is comprised of three distinct phases: the follicular phase (FP), the ovulatory phase, and the luteal phase (LP) [19, 20]. During the FP, estrogen is the dominant hormone, whereas progesterone prevails during the LP. These hormonal cycle phases are associated with numerous physiological and cognitive changes [21]. For example, Farage et al. [22] reviewed the effects of the MC on cognitive function and suggest that ovarian sex hormones mediate differences in cognition across various phases of the MC. In line with this, estrogen has been shown to generally improve cognitive performance, especially in tasks in which females outperform males such as verbal facility and memory [21, 22, 23]. Elevated estrogen levels, as measured via blood samples, are associated with enhanced fine motor dexterity [22, 24]. Whereas progesterone, sometimes associated with reduced cognitive performance [25], may also support memory function, as suggested by improvements in attention, verbal, and visual memory during the mid‐LP [22]. Spatial abilities, including mental rotations, show the opposite pattern: they show a strong negative correlation with estrogen levels, with the lowest scores (i.e., worst performance) observed during the mid‐LP and the highest (i.e., best performance) during menstruation [22, 24]. In contrast, females exhibit improved accuracy in spatial navigation during the LP (3–10 days after ovulation) compared to the early FP (1–6 days after menstruation onset) [26].

Therefore, hormonal fluctuations during MC contribute to cognitive function and may support the idea that ovarian sex hormones play a role in influencing both the severity and presentation of concussion‐related outcomes [7, 9, 10, 22]. In addition to these findings, several animal studies have supported the hypothesis that estrogen and progesterone can influence the neural outcomes of mTBI [27, 28], showing that these hormones can have varying effects on subsequent neural impairment. In some cases, estrogen has been observed to have neuroprotective effects, whereas in other instances, it has been shown to potentially exacerbate injury [29]. Research suggests that higher circulating levels of progesterone may offer neuroprotective benefits following an mTBI [30, 31] as well as supporting brain repair and recovery [31], with evidence pointing to its role in modulating inflammatory responses and promoting a neural environment conducive to healing [32]. However, evidence remains inconclusive, with conflicting results from human and animal studies highlighting the complexity of hormone interactions and their diverse effects on neural outcomes.

Besides these complexities regarding hormonal fluctuations in relation to concussion, it is crucial to critically assess current concussion evaluation protocols to ensure that they accurately account for individual differences and provide reliable outcomes. First, diagnosing a concussion requires a multidimensional approach [1] utilizing cognitive assessments through neuropsychological and neurocognitive testing such as the Immediate Postconcussion Assessment and Cognitive Test, standardized symptom scales, including the Postconcussion Symptom Scale, and postural stability checks for effective evaluation [33]. Each of these measures provides distinct and essential insights, because no single tool has the sensitivity to fully capture the complexity of concussion on its own [33, 34]. Even if multidimensional test protocols are carried out, another problem is that current testing protocols have certain limitations: They often fail to account for the disconnect between clinical symptoms and actual neurophysiological recovery, lack standardized assessment methods, and have no objective metrics to guide safer return‐to‐play (RTP) decisions [35]. Another significant limitation is the challenge of symptoms and their reporting. Notably, symptoms such as irritability, headache, difficulty concentrating, anxiety, and depression, which are often linked to concussion, can also be affected by hormonal fluctuations across the MC [22, 36]. The broad and nonspecific nature of these symptoms, which are not unique to concussion, along with their overlap with other conditions can compromise diagnostic accuracy, especially when they fluctuate over time [10]. Recognizing the overlap between postconcussion symptoms and those linked to the MC is essential for improving diagnostic precision and understanding the underlying causes [10].

To summarize, hormonal fluctuations during the MC have been shown to affect cognitive function, and MC‐related symptoms significantly overlap with postconcussion symptomatology. This highlights the need for a comprehensive investigation into the interaction between these factors. Current research underscores areas for further investigation, and particularly the role of hormonal phases in shaping outcomes following a concussion. The objective of this scoping review is to provide a comprehensive overview of the existing research on how MC phases influence concussion‐related outcomes. This includes one main question: whether the MC phase—both at the time of injury and after injury—influences postconcussion outcomes and symptom reporting. Additionally, building on current literature evaluating existing concussion testing protocols (for an overview, see [35]), another question examineshow the MC phase influences baseline assessments of concussion in the absence of injury. By addressing these questions, the review aims to consolidate current knowledge, identify gaps in research, and provide insights into how hormonal factors might inform more targeted approaches to concussion management and recovery protocols for female athletes.

## 2 Methods

This scoping review was conducted in accordance with the Preferred Reporting Items for Systematic Reviews and Meta‐Analyses extension for Scoping Reviews (PRISMA‐ScR) Statement guidelines [37]. It included all case–control, prospective cohort, nested cohort, and repeated measures observational studies, as well as single‐blinded prospective repeated measures designs that examined the MC, postconcussion outcomes, and symptom reporting.

This review included studies examining the effect of different MC phases (e.g., FP, LP) on postconcussion outcomes and symptom reporting, as well as studies examining baseline assessments of concussions in nonconcussed individuals in relation to the MC. The studies had to consider an MC phase and, if feasible, measure hormone levels directly. In the absence of direct measurement, an inference should be drawn based on the MC phase. The target population for this review was naturally menstruating females with self‐reported regular MCs (e.g., menses every 21–35 days). Studies including participants with irregular MCs, hormonal contraceptive (HC) users, nonusers, or comparisons between females and males were eligible for inclusion provided that these groups were clearly separated in the analysis. Importantly, the primary focus had to remain on outcomes in naturally menstruating females, in line with the aim of isolating the influence of MC phase on concussion‐related outcomes or baseline assessments.

### 2.1 Search Strategies

The following electric databases were searched from July to December 2024: Pubmed, Web of Science (all databases), and Federal Institute of Sports Science Germany (BISp SURF). The title, abstract, keywords, and full text were first examined. The strategy employed in all databases was identical. Further details regarding search strategies used for each database are as follows: (“sexual hormones” OR “hormones” OR “progesterone” OR “estradiol” OR “menstrual cycle” OR “menstrual phase”) AND (“concuss*” OR “mild traumatic brain injury” OR “mTBI” OR “traumatic brain injury” OR “TBI”) AND (“symptoms” OR “postconcussion” OR “symptom reporting”).

### 2.2 Selection Process

The studies included in this review were written in either English or German and their full text was available. Only original publications involving human participants and published between January 1980 and December 2024 were considered.

A screening strategy was utilized to identify and select studies for inclusion in this review, with Rayyan (http://rayyan.qcri.org) used to organize and screen studies throughout the review process [38]. The initial stage of the process involved screening the titles of the studies for relevance, followed by a check for duplicates. Duplicate studies found in the different databases were removed. The remaining abstracts were then evaluated against preestablished inclusion and exclusion criteria to identify relevant articles. Those deemed suitable based on the title and abstract review were subjected to a comprehensive full‐text screening conducted in accordance with the established criteria. The involvement of two reviewers ensured the reliability and consistency of the selection process.

## 3 Results

### 3.1 Study Selection

**FIGURE 1:**
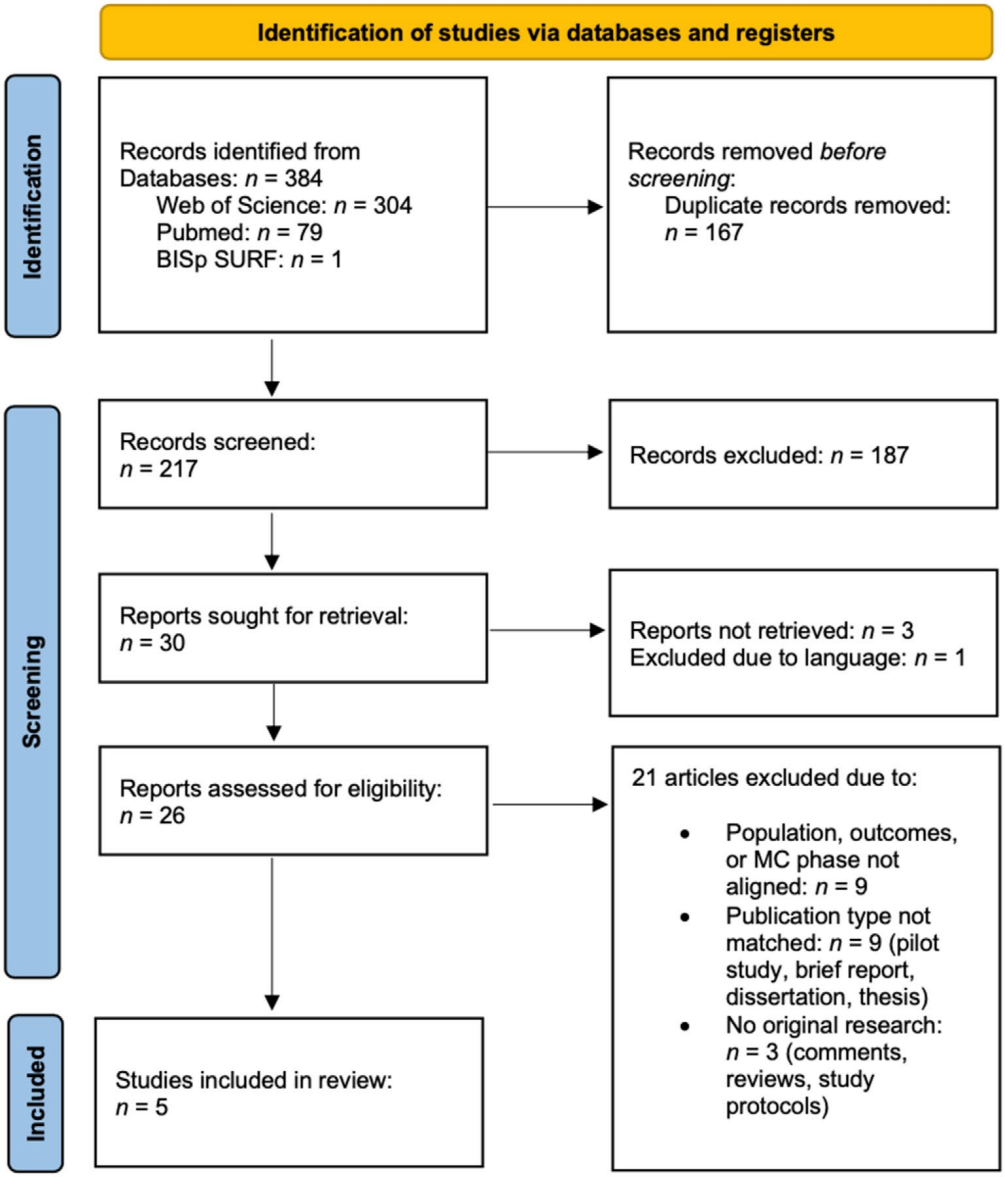
Preferred reporting items for systematic reviews and meta‐analyses extension for scoping reviews (PRISMA‐ScR) figure describes the pipeline of the selection process of included studies.

A total of 384 studies were identified (Figure 1). After removing duplicates, 217 studies proceeded to the screening stage. Following the screening of titles and abstracts, 30 studies were deemed eligible for full‐text review. However, three studies could not be retrieved, and one was excluded due to not meeting the language criteria, resulting in 26 studies assessed in full text. Ultimately, five studies were included in the final analysis [39, 40, 41, 42, 43].

During the screening process, nine studies were excluded because they did not sufficiently align with the main focus of the review. Specifically, these studies either examined the influence of HC use on baseline assessments or concussion‐related outcomes without considering MC phase, or they did not account for MC phase in their final analysis. In both instances, the MC phase was not a component of the stated research objectives and therefore did not meet the inclusion criteria. Additionally, nine studies were excluded because their publication type did not meet the inclusion criteria. Additionally, three studies were excluded as they were not original research articles. The complete selection process is illustrated in Figure 1.

### 3.2 Study Characteristics

Overall, this review included five studies investigating the relationship between MC phases and postconcussion outcomes and symptom reporting [39, 40, 41, 42, 43]. Ott et al. [41] evaluated whether neurocognitive performance and symptom experience following a sports‐related concussion in adolescent female athletes are influenced by MC‐related hormone levels. Roby et al. [42] and Wunderle et al. [43] investigated whether the MC phase at the time of injury could predict postconcussive symptoms. Mihalik et al. [40] explored how hormonal fluctuations during the MC may contribute to baseline symptom variability in nonconcussed individuals, whereas Malleck et al. [39] investigated this phenomenon specifically among HC users and nonusers, also focusing on nonconcussed individuals.

The studies, conducted between 2009 and 2024, encompassed a total of 774 participants aged 12–60 years. All participants were of reproductive age, postmenarcheal, and self‐reported regular MCs. Participants who did not self‐report regular MCs were assigned to a separate group by the respective studies. Furthermore, participants who were using HC were also placed in a different comparison group. The study designs included a case–control study, a single‐blinded prospective counterbalanced repeated measures design, a nested and prospective cohort study, and a repeated measures observational study.

**FIGURE 2:**
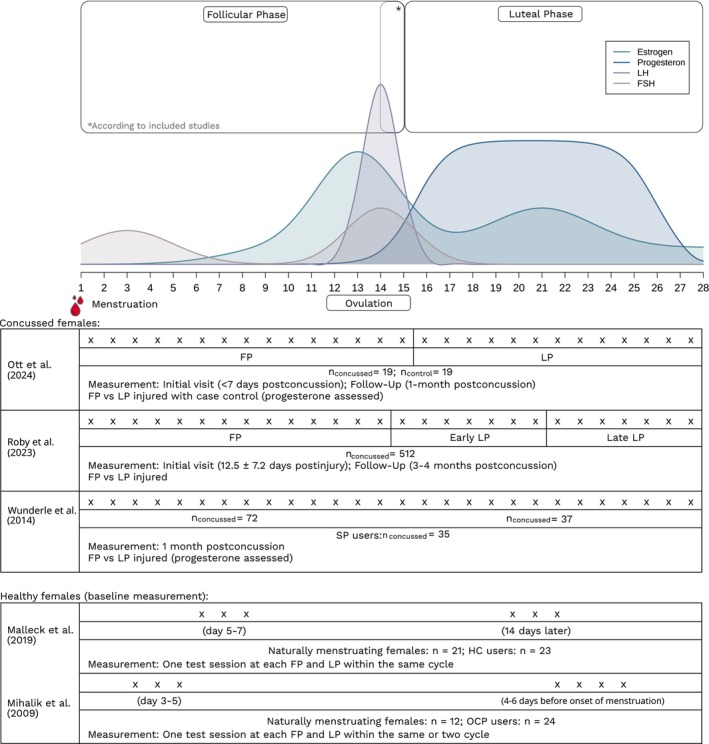
Overview of hormonal fluctuations and phases throughout menstrual cycle, along with the characteristics of the included studies FP, follicular phase; HC, hormonal contraceptives; LP, luteal phase; MC, menstrual cycle; OCP, oral contraceptive pills; SP users, synthetic progestin users.

All five studies considered both the FP and LP of the MC [39, 40, 41, 42, 43], with one study additionally differentiating between early and late LP [42]. Furthermore, Roby et al. [42] delineated menstruation as a discrete phase of the MC. Two studies measured the hormone progesterone via plasma blood samples [41] or serum blood samples [43]. For a more comprehensive overview, see Figure 2.

To assess postconcussion outcomes and symptom reports, the studies utilized a range of tools. The most frequently used testing protocols were the Immediate Postconcussion Assessment and Cognitive Test (ImPACT) [40, 41] and the Postconcussion Symptom Scale (PCSS) [39, 40, 41]. Some studies incorporated other measures such as the Depression, Anxiety, and Stress Scale (DASS) [39] and the Postconcussion Symptom Inventory (PCSI) [42]. Wunderle et al. [43] applied the Rivermead Postconcussion Questionnaire (RPCQ) alongside the EuroQoL/EQ5D, while Mihalik et al. [40] also used the Sensory Organization Test (SOT).

Follow‐up durations varied across the studies. Malleck et al. [39] and Mihalik et al. [40] evaluated outcomes at two points within the same MC. Ott et al. [41] conducted an initial assessment within 14 days postconcussion and a follow‐up assessment at the 6‐month mark. Roby et al. [42] measured the outcomes initially within 24 h postconcussion and again at 6 months. Wunderle et al. [43] concentrated their analysis on outcomes occurring 1 month after injury.

Across the reviewed studies, results show an influence of MC phases on symptom severity and health outcomes, with LP consistently associated with greater symptom exacerbation [41, 42, 43] and FP associated with more favorable or stable outcomes [42, 43]. Table S1 and Figure 2 provide further insights.

## 4 Discussion

This scoping review aimed to examine the influence of MC phases on postconcussion outcomes and symptom reporting by addressing one main question to identify existing gaps in knowledge about the MC and concussion‐related outcomes: whether the MC phase—both at the time of injury and after injury—influences postconcussion outcomes and symptom reporting. In addition to this, another key question addresses the overlap of MC symptoms and concussion testing protocols: how the MC phase influences baseline assessments of concussion in the absence of injury.

### 4.1 Concussion‐Related Outcomes and MC Phase

The results from the selected and reviewed studies suggest that hormonal fluctuations associated with the MC may influence postconcussion outcomes and symptom severity, supporting the notion that hormones play an important role in shaping both the severity and presentation of concussion‐related outcomes [41, 42, 43].

Notably, the MC phase at the time of injury has emerged as a potential predictor of postconcussion outcomes, a concept encapsulated in the “withdrawal hypothesis” proposed by Wunderle et al. [43] Specifically, if a mTBI occurs during the LP when progesterone levels are high, the sudden decline in this hormone at the end of this phase may result in more adverse clinical outcomes [43]. This decline in progesterone is regarded as a pivotal factor in the inferior outcomes observed in females relative to those observed in males who exhibit lower preinjury progesterone levels [43], supporting the notion that progesterone might have a neuroprotective effect that is lost following injury. Roby et al. [42] identified a trend toward lower symptom endorsement at follow‐up among females injured during the FP compared to those injured during the LP, proposing potentially worse outcomes for injuries sustained in the LP. However, this trend did not reach statistical significance, which limits the strength of this conclusion. Similarly, Ott et al. [41] reported greater symptom severity among females injured during the LP, further supporting the “withdrawal hypothesis” and suggesting that its neuroprotective effects may be lost as levels decline during the MC. These findings suggest that better outcomes during the FP may reflect a neuroprotective effect, while worse outcomes during the LP may be linked to the sudden drop in progesterone following injury.

In addition to the phase at the time of injury, the MC phase *following* a concussion may also influence the severity and presentation of concussion‐related outcomes. For example, symptom severity after concussion, particularly in the physical and cognitive domains, was higher during the LP when progesterone levels peak compared to the FP, when estrogen levels are higher. However, no significant effects of the MC phase were observed on neurocognitive indices such as visual memory, motor speed, or reaction time. At later follow up, symptom severity and neurocognitive outcomes were similar between LP and FP, indicating recovery over time regardless of the MC phase [41].

This connection between hormonal fluctuations and brain recovery has been further supported by research exploring the neuroprotective roles of ovarian sex hormones, particularly estrogen and progesterone, on brain health. Most other studies focused on TBI [32], the more severe form of traumatic brain injury [44]. Although the therapeutic potential of synthetic estrogen in TBI treatment has been studied, findings are based primarily on animal studies [13, 31]. Estrogens provide neuroprotection through various mechanisms following a TBI [32] including brain recovery by promoting cell survival, reducing damage from inflammation and oxidative stress, and fostering an environment conducive to healing [28]. These neuroprotective effects are mediated through estrogen receptors that regulate inflammation, promote neural repair, improve blood vessel health, and support the repair of neural connections [28, 45]. By preventing harmful cell death and balancing inflammatory responses (reducing proinflammatory signals and enhancing antiinflammatory ones), estrogen further mitigates damage and promotes long‐term recovery [46]. Whereas preclinical studies (in animals) have consistently shown these benefits, clinical trials in humans have not demonstrated the same robust effects [32]. This highlights the need for further research in order to gain a better understanding of, for example, differences between natural estrogen activity and synthetic treatments [32].

Building on these findings about the role of estrogens in TBI treatment, it is also worth exploring the potential contributions of progesterone. The administration of progesterone prior to brain injury has been demonstrated to reduce damage and improve recovery in animal studies [27, 47]. Similarly, clinical trials in humans with TBI have demonstrated that progesterone treatment can enhance recovery outcomes, with preclinical studies suggesting its potential to reduce neuroinflammation and prevent neuronal loss [31]. This neuroprotective effect is probably the result of progesterone's ability to stabilize brain pathways, support neural repair, and reduce excitotoxicity [31]. This hypothesis is supported by the findings of a study conducted by Chen et al. [30] that demonstrate that elevated circulating progesterone levels exert a protective effect following a concussion in college‐aged athletes. Although some studies have associated progesterone with negative effects in certain contexts [25], other research has indicated its potential for enhancing neural outcomes and promoting recovery when administered in a therapeutic context [31]. Recent studies have indicated that the progesterone‐to‐estradiol (P/E) ratio may potentially exert a more nuanced influence on the outcomes of concussion. For instance, a study by Goeckner et al. [48] specifically examined this ratio and found that a lower P/E ratio was associated with more severe psychological symptoms following concussion. The authors also emphasized that further research is needed to better understand and confirm the role of the P/E ratio in postconcussion outcomes [48].

### 4.2 Postconcussion Baseline Assessment and MC Phase

Given the issues regarding concussion protocols and diagnostic tools, evaluating systematic changes in postconcussion symptoms that are unrelated to the initial injury is crucial for improving concussion recovery evaluations in females. The substantial overlap between postconcussion symptoms and those associated with the MC makes it essential to recognize potential variations in symptoms throughout the MC in order to differentiate accurately their causes and assess their origin [39]. Furthermore, considering the MC during baseline testing is essential because Malleck et al. [39] have demonstrated that the presentation of symptoms varies significantly across MC phases in naturally menstruating females. If an athlete is measured in a different MC phase in the preseason baseline test than in the postinjury test, there is a risk that she will be cleared to play again prematurely [40]. Contrary to some assumptions about the influence of the MC on baseline testing, Mihalik et al. [40] found no interaction between MC phase and neuropsychological or postural stability test scores. This indicates that impairments observed in postinjury postural stability tests are more probably attributable to the concussion itself rather than being influenced by the MC phase. This finding underscores the importance of distinguishing concussion‐specific impairments from those influenced by hormonal fluctuations. Notably, Malleck et al. [39] highlighted that symptoms such as nausea and dizziness are more prevalent during the FP and could influence the testing outcome. These findings align with evidence showing that fluctuating ovarian sex hormones during the MC affect brain regions, leading to changes in cognitive and emotional processes throughout the MC. These hormonal fluctuations have been linked to changes in anxiety, depression, and somatic symptoms [36]. In particular, estrogen and progesterone play a key role in modulating mood and cognitive function, with effects including difficulty concentrating, irritability, and headaches—symptoms that are often assessed during baseline testing of athletes [22, 36]. Moreover, these hormones are also thought to affect emotion and cognition through dopaminergic pathways, with varying levels having different effects on brain structures and symptoms throughout the MC [36].

The impact of these hormonal fluctuations on symptom reporting is further supported by evidence highlighting sex‐based differences in baseline assessments. A meta‐analysis conducted by Brown et al. [10] identified significant differences in symptom reporting between males and females during baseline assessments, with females being 43% more likely to report symptoms linked to concussion. Furthermore, females were notably more prone to report specific symptoms, including headache, difficulty concentrating, vision and hearing issues, emotional disturbances, and problems with energy or sleep. However, these symptoms, frequently reported by females at baseline, are not necessarily unique to concussion and may instead reflect other physiological factors. For instance, symptoms such as headache, concentration difficulties, emotional fluctuations, and fatigue are often associated with premenstrual syndrome [10]. Additionally, Roffler et al. [49] highlight the diversity and frequency of MC symptoms in elite female athletes, noting that symptoms such as disturbed sleep, fatigue, bloating, and emotional changes were prevalent across different phases of the MC.

Taken together, these findings highlight the need to account for MC phases and hormonal fluctuations in baseline assessments, because they can influence symptom reporting and ensure accurate concussion evaluations, ultimately preventing premature RTP decisions.

### 4.3 General Discussion and Limitations

Overall, findings of this review underline the importance of the MC and its measurement, e.g., via tracking, in understanding and managing a concussion in females. However, this review identified only five studies, thereby highlighting the limited research available and the critical need for further investigation into the interplay between hormonal fluctuations during MC and concussion‐related outcomes.

Our findings suggest that hormonal fluctuations, particularly elevated progesterone during the LP, may intensify postconcussion symptoms and influence outcomes, thereby supporting the “withdrawal hypothesis” [42, 43]. However, research in this area is generally limited, with most findings derived from studies on TBI or animal models [13, 31, 32]. Nevertheless, these studies have inspired related clinical trials in humans and laid a foundation for exploring the therapeutic potential of hormonal interventions in mTBIs [30]. In a therapeutic context, estrogen and progesterone have shown potential neuroprotective effects by supporting brain recovery through mechanisms such as promoting cell survival [28], reducing inflammation, and enhancing recovery outcomes [31]. Building on this, the MC phase at the time of injury may also influence outcomes, with LP injuries potentially resulting in greater symptom severity, possibly due to the neuroprotective withdrawal of progesterone [41, 42, 43]. These hormonal fluctuations across the MC not only affect postconcussion outcomes but also influence symptoms such as headache and dizziness, which overlap with postconcussion symptoms, further complicating baseline concussion evaluations [39].

Since the potential neuroprotective roles of ovarian sex hormones in the treatment of TBI have been studied extensively in animal models [13, 31], is important to note that the application of these findings to human studies is complicated by differences in injury severity, brain structure, and the limited ability to assess subjective symptoms in animal models [13]. Although controlled animal models help to reduce the impact of individual variability, they have notable limitations. Injuries in animal studies are often more severe than those typically observed in human mTBI [50], and the measurement of functional outcomes lacks the sensitivity needed to capture subjective symptoms. Nonetheless, these animal studies have advanced the hypothesis that estrogen and progesterone may have neuroprotective effects, and this has inspired related clinical trials in humans [13, 30, 31].

While these insights advance our understanding, the studies included in this review face several limitations that impact their reliability and broader applicability. A major challenge is the transient nature of mTBI symptoms that may not manifest immediately and vary considerably between individuals and injury types, thereby complicating consistent cognitive assessments [3]. Additionally, the variability in injury mechanisms further complicates the analysis, because not all sustained concussions in the studies were sports‐related, and in some studies, the cause of injury was not clear [42, 43].

Another limitation is the potential inaccuracies arising from the reliance on self‐reported MC data instead of objective measures such as blood samples [39, 40, 42]. This issue is particularly pronounced in adolescents who often have irregular cycles and less experience in accurately reporting their MC phases, thereby further reducing data reliability [41, 42, 51, 52]. Additional limitations arise from small sample sizes [40, 41, 43], with Wunderle et al. [43] noting that the smaller sample sizes in the LP and progestin groups may have hindered the ability to achieve statistical significance.

However, these findings are not limited to concussed individuals. Two reviewed studies investigated healthy (i.e., nonconcussed) naturally menstruating females and found that symptoms such as nausea, dizziness, and difficulty falling asleep were more common during the FP and correlated with depression scores [39]. Despite these differences, the MC phase showed no significant effect on overall symptom severity, number of symptoms, or neurocognitive outcomes in healthy populations [40]. Taken together, these findings highlight the interplay between MC phases and symptomatology in both concussed and nonconcussed females. In addition, the reviewed studies also examined females using HC [39, 40]. Although it was not the primary focus of our research questions, results suggest that HC may also influence concussion‐related outcomes. This raises a new question: Could HC play a role in mitigating concussion symptoms in females? By stabilizing hormonal fluctuations that might otherwise exacerbate symptoms, HC could potentially reduce concussion‐related symptom severity by lowering circulating reproductive hormone levels [40].

Evidence suggests that HC use may reduce MC‐related variability, as demonstrated by greater consistency in symptom reporting among HC users compared to naturally menstruating females [39]. Furthermore, Mihalik et al. [40] found lower symptom severity among oral contraceptive users, whereas Wunderle et al. [43] observed improved outcomes in females using progestin–potentially due to the prevention of progesterone withdrawal.

However, other research presents conflicting findings on the impact of HC on concussion‐related outcomes: Some data indicate no differences in postconcussion symptom severity between HC users and nonusers [53], while other findings suggest reported lower symptom severity among HC users [15]. Moreover, there are also studies suggesting that HC does not significantly influence recovery trajectories [15, 53, 54]. Other studies suggest that worsened concussion symptoms stem from HC‐related increases in stress and inflammatory markers, which may counteract the neuroprotective effects of hormone stabilization [55].

Female athletes using HC have shown elevated stress and inflammation levels, possibly influencing recovery under high‐pressure conditions such as those in college sports [55]. Objective cognitive tests such as the ImPACT show no postconcussion cognitive differences between HC users and nonusers [53]. However, other research indicates enhanced cognitive functions in healthy (i.e., nonconcussed) HC users, including improvements in working memory, concentration [56], verbal memory [57], and visuospatial skills [58].

Given these conflicting findings and the complexity of hormonal influences, further research is needed to clarify the role of HCs in concussion‐related outcomes, especially considering its clinical relevance and growing importance.

### 4.4 Recommendations

Based on the results of this review and the given limitations, several recommendations can be made for future research and practice. Addressing these gaps is particularly important because the number of females engaging in sports and other high‐risk activities continues to grow, making it essential to gain a deeper understanding of how a patient's sex influences the outcome of mTBI [11]. Yet, females continue to be underrepresented in concussion studies, thereby limiting the understanding of sex‐specific risk factors, symptoms, and recovery patterns [59]. This underrepresentation highlights a persistent challenge in the field of concussion research among athletes: a significant sex imbalance [60, 61]. This disparity underscores an urgent need for action to address this gap in both research and (clinical) practice. This is also highlighted by a systematic review [62] examining 161 studies to assess the representation of female athletes in research on concussion. The authors found that the majority of sports‐related concussion studies examined male participants (80.1%), with 40.4% excluding female participants altogether. Recent research offers encouraging opportunities to address this imbalance. For example, studies are exploring short noncoding ribonucleic acids (sncRNAs) as salivary biomarkers for concussions in females [63]. These biomarkers have shown the potential to provide objective insights into concussion pathophysiology [64]. However, previous research has focused predominantly on male participants, thereby limiting its applicability to females [64]. Because sncRNA expression is sex‐dependent in other central nervous system diseases, expanding studies to include females is essential to fully understand their potential as diagnostic tools [65]. In addition to sncRNAs, blood‐based biomarkers such as Glial Fibrillary Acidic Protein (GFAP) and Ubiquitin C‐terminal hydrolase (UCH‐L1) are gaining traction as promising tools for improving diagnostic accuracy and offering objective insight into concussion beyond subjective symptom reports [66]. Nevertheless, these biomarker levels have been shown to vary by sex, potentially due to hormonal or tissue‐specific factors, although direct hormonal modulation has yet to be confirmed [67]. These findings further underscore the importance of including female participants in biomarker research, given that sex‐based physiological differences may influence both baseline levels and responses to injury. Despite some similarities between the sexes, physiological, biomechanical, and neuroanatomical differences can greatly influence concussion treatment and recovery [7, 16, 68]. Therefore, investigating more females is highly recommended.

As previously noted, there is significant overlap between postconcussion symptoms and those associated with the MC. To accurately identify the underlying causes of these symptoms and improve assessment accuracy, it is crucial to account for potential variations throughout the MC [39]. Baseline assessments conducted during one phase may not accurately reflect symptom severity during another, thereby complicating postconcussion evaluations [39]. Accordingly, it may be beneficial to conduct baseline assessments across multiple phases of the MC to capture a more comprehensive symptom profile and potential fluctuations in cognitive functions [69]. To address this, MC tracking should be integrated at baseline and throughout the recovery process, using methods such as symptom journaling, mobile tracking apps, or hormonal assessments [70, 71, 72]. Incorporating menstrual history and cycle tracking into concussion assessments for females is recommended to ensure a comprehensive evaluation [72]. MC tracking in research often involves apps such as FitrWoman [73], which has been used in previous studies and provides a calendar overview of past and current cycle phases [49]. However, most studies rely on a combination of methods—such as counting days, basal body temperature, menstrual symptoms, and cycle history [71]. In addition to these approaches, a recent review highlights salivary P4 and E2 (i.e., progesterone and estrogen) testing as a valid, promising, and noninvasive alternative to blood sampling for tracking menstrual hormones, especially in healthy premenopausal women, while recommending further large‐scale validation [70]. This approach may hold particular value in research and clinical settings focused on concussion, especially given the observed influence of the P/E ratio on postconcussion outcomes. Lower P/E ratios have been associated with more severe psychological symptoms in the acute phase [48]. These findings underscore the significance of monitoring not only individual hormone levels, but also their equilibrium. They further highlight the necessity for additional research investigating the impact of ovarian sex hormones, and their ratio, on concussion symptoms and outcomes.

Beyond its implications for concussion evaluations, there is increasing evidence to support the value of tracking the MC for exercise performance. Research, including a meta‐analysis by McNulty et al. [71], suggests that exercise performance may vary across different phases of the MC. Based on this evidence, there is already a recommendation for practitioners working with (elite) athletes to incorporate MC considerations into their athletic performance strategies. Practitioners are encouraged to recognize performance fluctuations across the MC and adopt personalized approaches to optimize training and competition outcomes [71]. Furthermore, Roffler et al. [49] highlight the critical role of recognizing individual symptom patterns throughout the MC in elite female athletes. By incorporating MC tracking and accounting for individual variability, practitioners may be able to improve diagnostic accuracy and provide more personalized care to female athletes.

**FIGURE 3:**
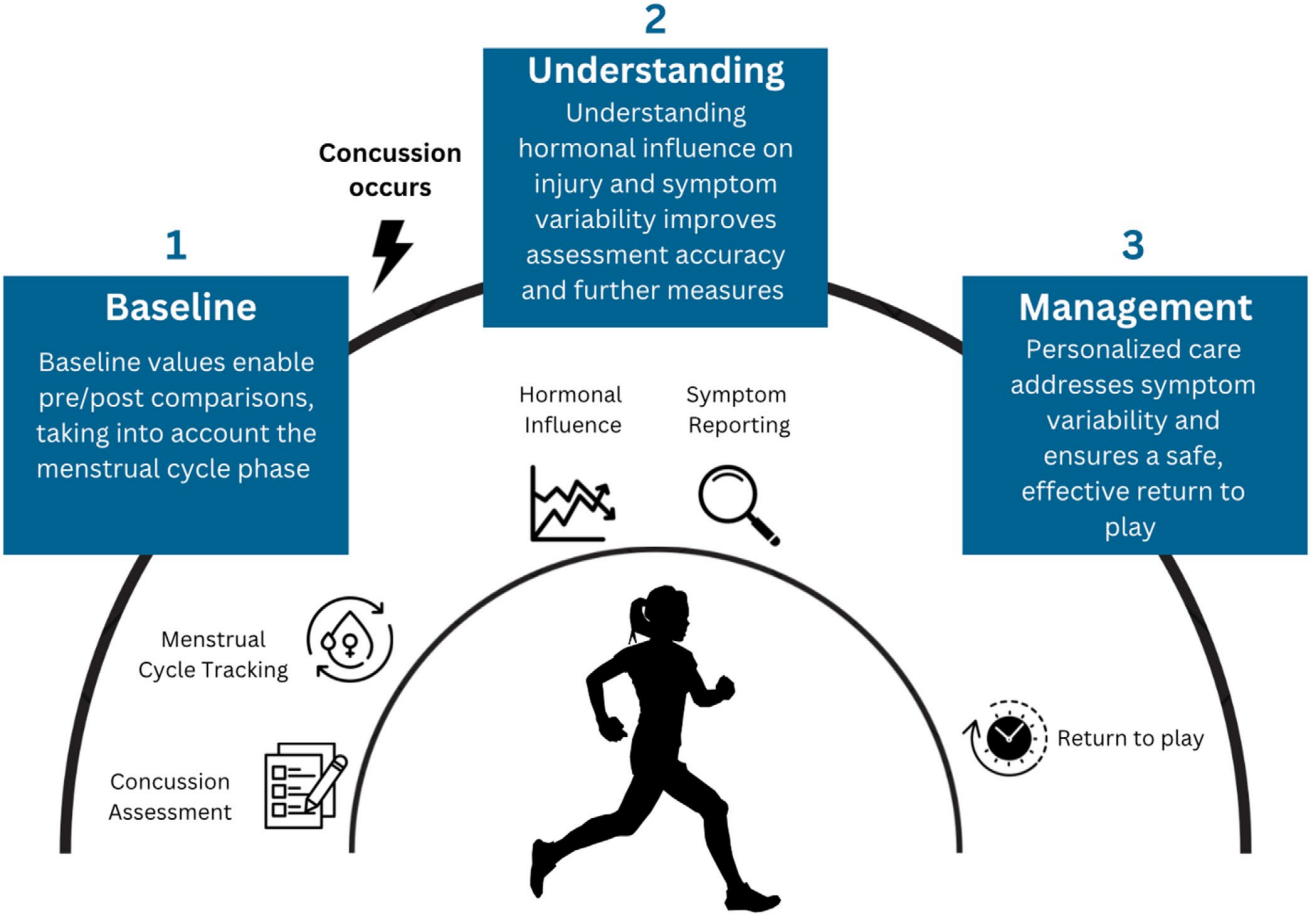
Recommendations for future research priorities in females: From baseline (1) and understanding (2) to management (3).

Finally, it is essential to integrate *baseline assessment data* (1), as seen in Figure 3, including both concussion‐specific metrics and MC tracking, into an athlete's profile. This dual approach allows practitioners to gain deeper insights when a concussion occurs by enabling comparisons between pre‐ and postinjury values.

Further, our findings suggest that hormonal fluctuations associated with the MC may significantly influence symptom severity and outcomes following a concussion, highlighting the critical role of hormones in concussion‐related outcomes. This underscores the importance of advancing our *understanding* (2) of concussion mechanisms and the various factors that contribute to outcome variability. However, as highlighted in the limitations, the transient nature of mTBI symptoms presents a major challenge. These symptoms may not manifest immediately, and they often vary significantly between individuals, thereby complicating the consistency of cognitive assessments [3]. Furthermore, the variability of injury mechanisms further complicates analysis, if not all concussions share a similar etiology (i.e., sports‐related). By addressing these complexities, practitioners and researchers can better understand how variabilities such as hormonal fluctuations influence concussion‐related outcomes. This understanding enables a more nuanced interpretation of symptom variability, improving both the accuracy of assessments and the effectiveness of treatment plans. Additionally, it is important to consider the potential overlap between postconcussion symptoms and MC‐related symptoms [10, 39]. Due to the variability and frequency of MC symptoms across different phases of the cycle [39], it cannot be definitively ruled out that typical MC phase symptoms may act as a moderator for increased postconcussion symptom reporting during certain phases. Furthermore, since baseline assessments conducted during one phase may not accurately reflect symptom severity in another [39], individuals who regularly experience symptoms during phases like the LP may perceive concussion symptoms as more severe due to additive effects. In order to more effectively isolate the effects of postconcussion symptoms, it is crucial that future research incorporates control groups that assess MC symptoms in individuals who do not have a concussion.

Achieving effective concussion *management* (3) in female athletes requires combining insights from *baseline assessments* and a deeper *understanding* of individual variabilities to create more personalized and informed strategies (see Figure 3). By integrating data on concussion‐specific metrics with MC tracking, practitioners and researchers can account for both physiological and hormonal factors that influence symptom presentation and recovery trajectories. This comprehensive approach not only enhances the accuracy of assessments but also ensures that recovery strategies are tailored to the unique needs of each athlete. By tracking both concussion‐specific metrics and MC data, practitioners and researchers can create a holistic view of an athlete's condition, enabling better support during recovery. In addition, these strategies align with broader evidence suggesting that personalized care, which accounts for tracking hormonal fluctuations and performance variability, optimizes both health and athletic performance outcomes [49, 71]. The goal is to leverage this comprehensive understanding in order to improve symptom assessment, support brain recovery, and enhance long‐term outcomes, ensuring that athletes RTP safely and effectively.

However, while individualized approaches are gaining traction, current practices still rely on generalized standards. International consensus guidelines outline a stepwise RTP process with uniform timeframes for all athletes [1, 2]. These protocols do not differentiate between male and female athletes, despite well‐documented sex differences in concussion outcomes [7, 8, 9, 10, 11, 13, 14]. No formal guidelines currently account for sex, even as growing evidence suggests this limits recovery accuracy and effectiveness. A 2021 study of college athletes emphasized that RTP protocols remain sex‐neutral despite clear sex‐based differences in recovery [74]. In response, research has begun advocating for more individualized RTP approaches that consider sex, moving away from the one‐size‐fits‐all model [9, 72].

This need is especially urgent given the diagnostic challenges posed by overlapping symptoms [10, 39]. Since RTP decisions depend on symptom resolution, the broad and nonspecific nature of postconcussion symptoms—many of which overlap with MC‐related symptoms—can compromise diagnostic accuracy [10, 39]. Female athletes may never be fully asymptomatic, as symptoms like headache, fatigue, concentration difficulties, and emotional changes often occur with the MC [75]. As a result, mild symptoms unrelated to the injury may persist, complicating assessments of true recovery and RTP readiness.

Future research should focus on investigating the role of ovarian sex hormones across different MC phases in concussion and developing objective biomarkers, such as salivary or blood hormone levels, to improve accuracy in MC phase assessment. Given the underrepresentation of females in studies, it is even more important for future research to prioritize the inclusion of female participants in order to address knowledge gaps in sex‐specific risk factors, symptoms, diagnosis, and recovery patterns, but also prevention strategies (e.g., neck exercise as a risk reduction strategy) [76]. Moreover, refining baseline testing protocols to account for MC variability will enhance the accuracy of postinjury assessments in female athletes. These efforts are crucial not only to advance scientific knowledge but also to improve clinical practices, ensuring more equitable and effective care for female athletes.

## 5 Perspective

This review highlights the need to integrate MC phase considerations into concussion management in order to enhance the precision of assessments and the efficacy of recovery strategies in female athletes. The findings of the included studies underscore the influence of hormonal fluctuations on concussion‐related outcomes [41, 42, 43] and emphasize the significant overlap between postconcussion symptoms and MC‐related symptom variability [39]. Also, results highlight the importance of developing baseline testing protocols to account for MC phase variability, because this could improve diagnostic accuracy and recovery outcomes [39, 72]. Furthermore, this review contributes to ongoing discussions regarding the underrepresentation of female athletes in concussion research [59, 62]. Addressing this gap is critical to developing sex‐specific guidelines that integrate both physiological and hormonal factors into concussion assessment and treatment plans. Building on recent studies advocating personalized care strategies [49, 71], these findings reinforce the need to track MC phases to optimize health and performance outcomes in female athletes. Future research can enhance understanding of the influence of hormonal fluctuations on concussion outcomes and recovery, contributing to both scientific progress and practical applications in female athlete care.

## Conflicts of Interest

The authors declare no conflicts of interest.

## Supporting information


Data S1.

## Data Availability

Data sharing is not applicable to this article as no new data were created or analyzed in this study.

## References

[1] P. McCrory , W. Meeuwisse , J. Dvořák , et al., “Consensus Statement on Concussion in Sport—The 5th International Conference on Concussion in Sport Held in Berlin, October 2016,” British Journal of Sports Medicine 51, no. 11 (2017): 838–847, 10.1136/bjsports-2017-097699. 28446457

[2] P. McCrory , W. H. Meeuwisse , M. Aubry , et al., “Consensus Statement on Concussion in Sport: The 4th International Conference on Concussion in Sport Held in Zurich, November 2012,” Journal of Athletic Training 48, no. 4 (2013): 554–575, 10.4085/1062-6050-48.4.05. 23855364 PMC3715021

[3] N. J. Washnik , J. Anjum , K. Lundgren , and S. Phillips , “A Review of the Role of Auditory Evoked Potentials in Mild Traumatic Brain Injury Assessment,” Trends Hear 23 (2019): 233121651984009, 10.1177/2331216519840094. PMC6475843 30995888

[4] K. G. Harmon , J. Drezner , M. Gammons , et al., “American Medical Society for Sports Medicine Position Statement: Concussion in Sport,” Clinical Journal of Sport Medicine 47, no. 1 (2013): 15–26, 10.1136/bjsports-2012-091941. 23243113

[5] A. S. Gonschorek , “Hart getroffen: Gehirnerschütterungen im Sport,” Trauma Und Berufskrankheit 20, no. Suppl 1 (2018): S64–S66, 10.1007/s10039-017-0299-x.

[6] L. Wu , Y. Li , M. Sun , P. Ye , Z. Zhang , and W. Liu , “Global, Regional, and National Burdens of Mild Traumatic Brain Injuries From 1990 to 2019: Findings From the Global Burden of Disease Study 2019 – A Cross‐Sectional Study,” International Journal of Surgery 111 (2025): 160–170, 10.1097/JS9.0000000000001837. 38913425 PMC11745685

[7] R. P. Gupte , W. M. Brooks , R. R. Vukas , J. D. Pierce , and J. L. Harris , “Sex Differences in Traumatic Brain Injury: What We Know and What We Should Know,” Journal of Neurotrauma 36, no. 22 (2019): 3063–3091, 10.1089/neu.2018.6171. 30794028 PMC6818488

[8] T. Covassin , C. B. Swanik , and M. L. Sachs , “Sex Differences and the Incidence of Concussions Among Collegiate Athletes,” Journal of Athletic Training 38, no. 3 (2003): 238–244. 14608434 PMC233178

[9] D. K. Broshek , T. Kaushik , J. R. Freeman , D. Erlanger , F. Webbe , and J. T. Barth , “Sex Differences in Outcome Following Sports‐Related Concussion,” Journal of Neurosurgery 102, no. 5 (2005): 856–863, 10.3171/jns.2005.102.5.0856. 15926710

[10] D. A. Brown , J. A. Elsass , A. J. Miller , L. E. Reed , and J. C. Reneker , “Differences in Symptom Reporting Between Males and Females at Baseline and After a Sports‐Related Concussion: A Systematic Review and Meta‐Analysis,” Sports Medicine 45 (2015): 1027–1040, 10.1007/s40279-015-0335-6. 25971368

[11] E. Farace and W. M. Alves , “Do Women Fare Worse? A Metaanalysis of Gender Differences in Outcome After Traumatic Brain Injury,” Neurosurgical Focus 8, no. 1 (2000): 1–8, 10.3171/foc.2000.8.1.152. 16924776

[12] R. N. Moran , J. R. Guin , J. Gardner , and J. Simer , “Baseline Computerized Neurocognitive Testing and Oculomotor Measures Are Not Altered by Hormonal Contraceptive Use,” Archives of Clinical Neuropsychology 38, no. 6 (2023): 922–928, 10.1093/arclin/acad015. 36759181

[13] J. J. Bazarian , B. Blyth , S. Mookerjee , H. He , and M. P. McDermott , “Sex Differences in Outcome After Mild Traumatic Brain Injury,” Journal of Neurotrauma 27, no. 3 (2010): 527–539, 10.1089/neu.2009.1068. 19938945 PMC2867588

[14] A. C. Colvin , J. Mullen , M. R. Lovell , R. V. West , M. W. Collins , and M. Groh , “The Role of Concussion History and Gender in Recovery From Soccer‐Related Concussion,” American Journal of Sports Medicine 37, no. 9 (2009): 1699–1704, 10.1177/0363546509332497. 19460813

[15] V. Gallagher , N. Kramer , K. Abbott , et al., “The Effects of Sex Differences and Hormonal Contraception on Outcomes After Collegiate Sports‐Related Concussion,” Journal of Neurotrauma 35, no. 11 (2018): 1242–1247, 10.1089/neu.2017.5453. 29336208 PMC10331147

[16] S. Stone , B. Lee , J. C. Garrison , D. Blueitt , and K. Creed , “Sex Differences in Time to Return‐To‐Play Progression After Sport‐Related Concussion,” Sports Health 9, no. 1 (2017): 41–44, 10.1177/1941738116672184. 27697890 PMC5315255

[17] M. Mountjoy , J. Sundgot‐Borgen , L. Burke , et al., “The IOC Consensus Statement: Beyond the Female Athlete Triad—Relative Energy Deficiency in Sport (RED‐S),” British Journal of Sports Medicine 48, no. 7 (2014): 491–497, 10.1136/bjsports-2014-093502. 24620037

[18] I. Soumpasis , B. Grace , and S. Johnson , “Real‐Life Insights on Menstrual Cycles and Ovulation Using Big Data,” Human Reproduction Open 2020, no. 2 (2020): hoaa011, 10.1093/hropen/hoaa011. 32328534 PMC7164578

[19] K. Itriyeva , “The Normal Menstrual Cycle,” Current Problems in Pediatric and Adolescent Health Care 52, no. 5 (2022): 101183, 10.1016/j.cppeds.2022.101183. 35527220

[20] T. Reilly , “The Menstrual Cycle and Human Performance: An Overview,” Biological Rhythm Research 31, no. 1 (2000): 29–40, 10.1076/0929-1016(200002)31:1;1-0;FT029. 11543399

[21] B. B. Sherwin , “Estrogen and Cognitive Functioning in Women: Lessons We Have Learned,” Behavioral Neuroscience 126, no. 1 (2011): 123–127, 10.1037/a0025539. 22004260 PMC4838456

[22] M. A. Farage , T. W. Osborn , and A. B. MacLean , “Cognitive, Sensory, and Emotional Changes Associated With the Menstrual Cycle: A Review,” Archives of Gynecology and Obstetrics 278, no. 4 (2008): 299–307, 10.1007/s00404-008-0708-2. 18592262

[23] E. Hogervorst , “Estrogen and the Brain: Does Estrogen Treatment Improve Cognitive Function?,” Menopause International 19, no. 1 (2013): 6–19, 10.1177/1754045312473873. 27951525

[24] P. M. Maki , J. B. Rich , and R. S. Rosenbaum , “Implicit Memory Varies Across the Menstrual Cycle: Estrogen Effects in Young Women,” Neuropsychologia 40, no. 5 (2002): 518–529, 10.1016/s0028-3932(01)00126-9. 11749982

[25] A. M. Warren , C. Gurvich , R. Worsley , and J. Kulkarni , “A Systematic Review of the Impact of Oral Contraceptives on Cognition,” Contraception 90, no. 2 (2014): 111–116, 10.1016/j.contraception.2014.03.015. 24856205

[26] A. Scheuringer and B. Pletzer , “Sex Differences and Menstrual Cycle Dependent Changes in Cognitive Strategies During Spatial Navigation and Verbal Fluency,” Frontiers in Psychology 8 (2017): 381, 10.3389/fpsyg.2017.00381. 28367133 PMC5355435

[27] E. H. Pettus , D. W. Wright , D. G. Stein , and S. W. Hoffman , “Progesterone Treatment Inhibits the Inflammatory Agents That Accompany Traumatic Brain Injury,” Brain Research 1049, no. 1 (2005): 112–119, 10.1016/j.brainres.2005.05.004. 15932748

[28] N. Raghava , B. C. Das , and S. K. Ray , “Neuroprotective Effects of Estrogen in CNS Injuries: Insights From Animal Models,” Neuroscience and Neuroeconomics 6 (2017): 15–29, 10.2147/nan.s105134. 28845391 PMC5567743

[29] R. L. Roof and E. D. Hall , “Gender Differences in Acute CNS Trauma and Stroke: Neuroprotective Effects of Estrogen and Progesterone,” Journal of Neurotrauma 17, no. 5 (2000): 367–388, 10.1089/neu.2000.17.367. 10833057

[30] Y. Chen , A. A. Herrold , V. Gallagher , et al., “Preliminary Report: Localized Cerebral Blood Flow Mediates the Relationship Between Progesterone and Perceived Stress Symptoms Among Female Collegiate Club Athletes After Mild Traumatic Brain Injury,” Journal of Neurotrauma 38, no. 13 (2021): 1809–1820, 10.1089/neu.2020.7217. 33470158 PMC8336258

[31] D. G. Stein , “A Clinical/Translational Perspective: Can a Developmental Hormone Play a Role in the Treatment of Traumatic Brain Injury?,” Hormones and Behavior 63, no. 2 (2013): 291–300, 10.1016/j.yhbeh.2012.05.004. 22626570

[32] K. A. Duncan , “Estrogen Formation and Inactivation Following TBI: What We Know and Where We Could Go,” Frontiers in Endocrinology 11, no. 345 (2020): 1–9, 10.3389/fendo.2020.00345. 32547495 PMC7272601

[33] S. P. Broglio , S. N. Nacciocchi , and M. S. Ferrara , “Sensitivity of the Concussion Assessment Battery,” Neurosurgery 60, no. 6 (2007): 1050–1057, 10.1227/01.NEU.0000255479.90999.C0. 17538379

[34] M. Aubry , R. Cantu , J. Dvorak , et al., “Summary and Agreement Statement of the 1st International Symposium on Concussion in Sport, Vienna 2001,” Physician and Sportsmedicine 30, no. 2 (2002): 57–63, 10.3810/psm.2002.02.176. 20086514

[35] D. Wellm and K. Zentgraf , “Diagnostic Tools for Return‐To‐Play Decisions in Sports‐Related Concussion,” Journal of Concussion 7 (2023): 1–18, 10.1177/20597002231183234.

[36] J. Sacher , H. Okon‐Singer , and A. Villringer , “Evidence From Neuroimaging for the Role of the Menstrual Cycle in the Interplay of Emotion and Cognition,” Frontiers in Human Neuroscience 7, no. 374 (2013): 1–7, 10.3389/fnhum.2013.00374. 23898247 PMC3721046

[37] A. C. Tricco , E. Lillie , W. Zarin , et al., “PRISMA Extension for Scoping Reviews (PRISMA‐ScR): Checklist and Explanation,” Annals of Internal Medicine 169, no. 7 (2018): 467–473, 10.7326/M18-0850. 30178033

[38] M. Ouzzani , H. Hammady , Z. Fedorowicz , and A. Elmagarmid , “Rayyan—A Web and Mobile App for Systematic Reviews,” Systematic Reviews 5, no. 1 (2016): 210, 10.1186/s13643-016-0384-4. 27919275 PMC5139140

[39] M. Malleck , K. J. Milne , and C. A. Abeare , “The Effect of Menstrual Cycle Phase and Hormonal Contraceptive Use on Post‐Concussive Symptom Reporting in Non‐Concussed Adults,” Psychological Injury and Law 12, no. 2 (2019): 183–190, 10.1007/s12207-019-09351-z.

[40] J. P. Mihalik , K. S. Ondrak , K. M. Guskiewicz , and R. G. McMurray , “The Effects of Menstrual Cycle Phase on Clinical Measures of Concussion in Healthy College‐Aged Females,” Journal of Science and Medicine in Sport 12, no. 3 (2009): 383–387, 10.1016/j.jsams.2008.05.003. 18771954

[41] S. Ott , J. Redell , S. Cheema , P. Schatz , and E. Becker , “Progesterone Levels in Adolescent Female Athletes May Contribute to Decreased Cognitive Performance During Acute Phase of Sports‐Related Concussion,” Developmental Neuropsychology 49, no. 2 (2024): 86–97, 10.1080/87565641.2024.2309556. 38314752

[42] P. R. Roby , A. Grimberg , C. L. Master , and K. B. Arbogast , “Menstrual Cycle Patterns After Concussion in Adolescent Patients,” Journal of Pediatrics 262 (2023): 113349, 10.1016/j.jpeds.2023.02.002. 36796579 PMC10423739

[43] K. Wunderle , K. M. Hoeger , E. Wasserman , and J. J. Bazarian , “Menstrual Phase as Predictor of Outcome After Mild Traumatic Brain Injury in Women,” Journal of Head Trauma Rehabilitation 29, no. 5 (2014): E1–E8, 10.1097/htr.0000000000000006. PMC5237582 24220566

[44] H. Hallock , M. Mantwill , P. Vajkoczy , et al., “Sport‐Related Concussion,” Neurology: Clinical Practice 13, no. 2 (2023): e200123, 10.1212/cpj.0000000000200123. 36891462 PMC9987206

[45] C. J. Saldanha , K. A. Duncan , and B. J. Walters , “Neuroprotective Actions of Brain Aromatase,” Frontiers in Neuroendocrinology 30, no. 2 (2009): 106–118, 10.1016/j.yfrne.2009.04.016. 19450619 PMC2700852

[46] N. L. Day , C. L. Floyd , T. L. D'Alessandro , W. J. Hubbard , and I. H. Chaudry , “17β‐Estradiol Confers Protection After Traumatic Brain Injury in the Rat and Involves Activation of G Protein‐Coupled Estrogen Receptor 1,” Journal of Neurotrauma 30, no. 17 (2013): 1531–1541, 10.1089/neu.2013.2854. 23659385 PMC3751264

[47] N. Jiang , M. Chopp , D. Stein , and H. Feit , “Progesterone Is Neuroprotective After Transient Middle Cerebral Artery Occlusion in Male Rats,” Brain Research 735, no. 1 (1996): 101–107, 10.1016/0006-8993(96)00605-1. 8905174

[48] B. D. Goeckner , D. L. Huber , K. Van Bortel , et al., “Progesterone and Estradiol Levels Associated With Concussion and Clinical Outcomes and Recovery in Female Athletes and Cadets,” Medicine and Science in Sports and Exercise 57, no. 3 (2025): 524–534, 10.1249/MSS.0000000000003591. 39501473 PMC11828682

[49] A. Roffler , M. T. Fleddermann , H. De Haan , K. Krüger , and K. Zentgraf , “Menstrual Cycle Tracking in Professional Volleyball Athletes,” Frontiers in Sports and Active Living 6 (2024): 1408711, 10.3389/fspor.2024.1408711. 39005625 PMC11239427

[50] R. R. Leker , E. Shohami , and S. Constantini , “Experimental Models of Head Trauma,” Acta Neurochirurgica. Supplement 83 (2002): 49–54, 10.1007/978-3-7091-6743-4_9. 12442621

[51] E. Deligeoroglou , N. Athanasopoulos , P. Tsimaris , K. D. Dimopoulos , N. Vrachnis , and G. Creatsas , “Evaluation and Management of Adolescent Amenorrhea,” Annals of the New York Academy of Sciences 1205, no. 1 (2010): 23–32, 10.1111/j.1749-6632.2010.05669.x. 20840249

[52] A. M. Z. Jukic , C. R. Weinberg , A. J. Wilcox , D. R. McConnaughey , P. Hornsby , and D. D. Baird , “Accuracy of Reporting of Menstrual Cycle Length,” American Journal of Epidemiology 167, no. 1 (2007): 25–33, 10.1093/aje/kwm265. 17928401 PMC3693484

[53] J. J. M. Kay , K. I. Mangold , A. Lapointe , et al., “Hormonal Contraceptives Do Not Influence Concussion Recovery in Collegiate Athletes: Data From the NCAA‐DoD CARE Consortium,” Medicine and Science in Sports and Exercise 55, no. 8 (2023): 1375–1381, 10.1249/mss.0000000000003162. 36897829

[54] H. C. Bouchard , P. M. Kelshaw , T. G. Bowman , et al., “Exploring the Relationship Between Contraceptive Medication Use and Concussion Recovery in Female Collegiate Athletes: A LIMBIC MATARS Consortium Investigation,” Brain Injury 9 (2024): 1–7, 10.1080/02699052.2024.2310780. 38335246

[55] B. N. Bozzini , B. A. McFadden , K. J. Elliott‐Sale , P. A. Swinton , and S. M. Arent , “Evaluating the Effects of Oral Contraceptive Use on Biomarkers and Body Composition During a Competitive Season in Collegiate Female Soccer Players,” Journal of Applied Physiology 130, no. 6 (2021): 1971–1982, 10.1152/japplphysiol.00818.2020. 33955263

[56] J. L. Holloway , K. D. Beck , and R. J. Servatius , “Facilitated Acquisition of the Classically Conditioned Eyeblink Response in Females Is Augmented in Those Taking Oral Contraceptives,” Behavioural Brain Research 216, no. 1 (2010): 301–307, 10.1016/j.bbr.2010.08.008. 20723566

[57] K. L. Mordecai , L. H. Rubin , and P. M. Maki , “Effects of Menstrual Cycle Phase and Oral Contraceptive Use on Verbal Memory,” Hormones and Behavior 54, no. 2 (2008): 286–293, 10.1016/j.yhbeh.2008.03.006. 18455727

[58] E. Cicinelli , M. De Tommaso , and A. Cianci , “Oral Contraceptive Therapy Modulates Hemispheric Asymmetry in Spatial Attention,” Contraception 84, no. 6 (2011): 634–636, 10.1016/j.contraception.2011.03.016. 22078194

[59] C. De Borja , C. J. Chang , R. Watkins , and C. Senter , “Optimizing Health and Athletic Performance for Women,” Current Reviews in Musculoskeletal Medicine 15, no. 1 (2022): 10–20, 10.1007/s12178-021-09735-2. 35023069 PMC8804053

[60] R. Edelstein , S. Gutterman , B. Newman , and J. D. Van Horn , “Assessment of Sports Concussion in Female Athletes: A Role for Neuroinformatics?,” Neuroinformatics 22, no. 4 (2024): 607–618, 10.1007/s12021-024-09680-8. 39078562 PMC11579174

[61] N. Nowatzki and K. R. Grant , “Sex Is Not Enough: The Need for Gender‐Based Analysis in Health Research,” Health Care for Women International 32, no. 4 (2011): 263–277, 10.1080/07399332.2010.519838. 21409661

[62] C. D'Lauro , B. R. Johnson , G. McGinty , C. D. Allred , D. E. Campbell , and J. C. Jackson , “Reconsidering Return‐To‐Play Times: A Broader Perspective on Concussion Recovery,” Orthopaedic Journal of Sports Medicine 6, no. 3 (2018): 1–7, 10.1177/2325967118760854. PMC5858632 29568786

[63] N. Hardaker , D. King , P. A. Hume , et al., “Female RNA Concussion (FeRNAC) Study: Assessing Hormone Profiles and Salivary RNA in Females With Concussion by Emergency Departments in New Zealand: A Study Protocol,” BMC Neurology 24, no. 1 (2024): 149, 10.1186/s12883-024-03653-9. 38698312 PMC11064333

[64] V. Di Pietro , P. O'Halloran , C. N. Watson , et al., “Unique Diagnostic Signatures of Concussion in the Saliva of Male Athletes: The Study of Concussion in Rugby Union Through microRNAs (SCRUM),” British Journal of Sports Medicine 55, no. 24 (2021): 1395–1404, 10.1136/bjsports-2020-103274. 33757972 PMC8639909

[65] M. Muñoz‐Culla , H. Irizar , M. Sáenz‐Cuesta , et al., “SncRNA (microRNA & snoRNA) Opposite Expression Pattern Found in Multiple Sclerosis Relapse and Remission Is Sex Dependent,” Scientific Reports 6, no. 1 (2016): 20126, 10.1038/srep20126. 26831009 PMC4735588

[66] M. McCrea , S. P. Broglio , T. W. McAllister , et al., “Association of Blood Biomarkers With Acute Sport‐Related Concussion in Collegiate Athletes: Findings From the NCAA and Department of Defense CARE Consortium,” JAMA Network Open 3, no. 1 (2020): e1919771, 10.1001/jamanetworkopen.2019.19771. 31977061 PMC6991302

[67] L. Papa , G. M. Brophy , W. Alvarez , et al., “Sex Differences in Time Course and Diagnostic Accuracy of GFAP and UCH‐L1 in Trauma Patients With Mild Traumatic Brain Injury,” Scientific Reports 13 (2023): 11833, 10.1038/s41598-023-38804-4. 37481589 PMC10363108

[68] E. Chamard , M. Lassonde , and L. Henry , “Neurometabolic and Microstructural Alterations Following a Sports‐Related Concussion in Female Athletes,” Brain Injury 27, no. 9 (2013): 1038–1046, 10.3109/02699052.2013.794968. 23834633

[69] F. Ronca , J. M. Blodgett , G. Bruinvels , et al., “Attentional, Anticipatory and Spatial Cognition Fluctuate Throughout the Menstrual Cycle: Potential Implications for Female Sport,” Neuropsychologia 206 (2025): 108909, 10.1016/j.neuropsychologia.2024.108909. 38762068

[70] T. Huang , F. M. Howse , N. S. Stachenfeld , and C. W. Usselman , “Correlations Between Salivary‐ and Blood‐Derived Gonadal Hormone Assessments and Implications for Inclusion of Female Participants in Research Studies,” American Journal of Physiology. Heart and Circulatory Physiology 324, no. 1 (2023): H33–H46, 10.1152/ajpheart.00399.2022. 36426884

[71] K. L. McNulty , K. J. Elliott‐Sale , E. Dolan , et al., “The Effects of Menstrual Cycle Phase on Exercise Performance in Eumenorrheic Women: A Systematic Review and Meta‐Analysis,” Sports Medicine 50, no. 10 (2020): 1813–1827, 10.1007/s40279-020-01319-3. 32661839 PMC7497427

[72] K. Stephenson , M. N. Womble , N. Hawa , and R. J. Elbin , “Clinical Considerations for the Assessment, Management, and Treatment of Concussion in Females,” Annals of Joint 6 (2021): 39, 10.21037/aoj-20-43.

[73] FitrWoman , “Period Tracking and Training App for Active Women,” accessed 7 April 2025, https://www.fitrwoman.com.

[74] E. E. Kieffer , P. G. Brolinson , A. E. Maerlender , E. P. Smith , and S. Rowson , “In‐Season Concussion Symptom Reporting in Male and Female Collegiate Rugby Athletes,” Neurotrauma Reports 2, no. 1 (2021): 503–511, 10.1089/neur.2021.0050. 34901945 PMC8655811

[75] A. Hassen , A. Klingaman , and J. C. Reneker , “Medical Management of Males and Females in Return to Learn and Return to Play After Concussion: An Observational Study,” International Journal of Sports and Exercise Medicine 3 (2017): 66, 10.23937/2469-5718/1510066.

[76] C. Müller and K. Zentgraf , “Neck and Trunk Strength Training to Mitigate Head Acceleration in Youth Soccer Players: Original Research,” Journal of Strength and Conditioning Research 35, no. 12S (2021): S81–S89, 10.1519/JSC.0000000000003863. 33065700

