# Supplementary material for: The Influence of Menstrual Cycle Phases on Postconcussion Outcomes and Symptom Reporting: A Scoping Review
Source: Scand J Med Sci Sports. 2025 Jun 25;35(7):e70093. doi: 10.1111/sms.70093 (PMC12188701; doi:10.1111/sms.70093)
Supplement: Supplementary file 1 — Data S1. [file SMS-35-e70093-s001.docx]

|  | | Supplementary Material:  Table 1. Overview of Study Characteristics and the Influence of Menstrual Cycle Phases on Concussion-related Outcomes. | | | | | | |  |  |
| --- | --- | --- | --- | --- | --- | --- | --- | --- | --- | --- |
| References | Study design | | Population | Menstrual phase considered | Measured hormones | Measured menstrual phase | Injury Cause | Assessments used | Timeline of concussion outcomes measurement | Key findings |
| Malleck et al. (2019) | Repeated measures observational study | | 44 healthy females (i.e., nonconcussed) 1. naturally menstruating (age 22.14 ± 4.13 years, n = 21) 2. HC users (age 21.30 ± 3.15 years, n = 23) (type of HC not specified) | Follicular (Days 5-7) and luteal phase (Days 19-21) | Not measured | Self-reported menstrual cycle phases | N/A | Post-Concussion Symptom Scale (PCSS) and Depression, Anxiety, and Stress Scale (DASS) | Two test sessions:  1. 5–7 days after onset MC (FP)  2. 14 days later (LP) within the same cycle | Naturally menstruating females showed changes in specific symptoms across MC, whereas females with HC did not show such changes. |
| Mihalik et al. (2009) | Single-blinded prospective counterbalanced repeated measures design | | 36 healthy females (i.e., nonconcussed), (age 21.4 ± 1.7 years) 1. OCP users (n = 24) 2. naturally menstruating (n = 12) | Follicular and luteal phase (no day range specified) | Not measured | Self-reported menstrual cycle phases | N/A | Immediate Post-Concussion Assessment and Cognitive Test (ImPACT), Post-Concussion Symptom Scale (PCSS) as part of ImPACT, Sensory Organization Test (SOT) | Two test sessions:  1. FP (early, 3-5 days after cessation of menstrual flow) 2. LP (late, 4-6 days prior to onset of menstrual flow) within the same or two cycles | No significant effects of menstrual phase on concussion measures (SOT, ImPACT). Naturally menstruating females reported higher symptom severity and more symptoms than OCP users. |
| Ott et al. (2024) | Case-control study | | 38 adolescent female athletes (i.e., concussed),  1. SRC group (age 15.95 ± 1.43 years, n = 19) 2. Control group (age 16,.05 ± 1.39 years, n = 19) | Follicular (Days 1-15) and luteal phase (Days after 15) | Progesterone levels assessed from blood samples (plasma) | Self-reported menstrual cycle phases | Injury cause not specified | Post-Concussion Symptom Scale (PCSS) as part of ImPACT | Initial visit (<7 days postconcussion), Follow up (1-month postconcussion) | Higher progesterone levels were associated with higher postconcussion symptom scores in the LP. Concussed females showed significantly lower verbal memory scores compared to controls. By Visit 2, no significant differences in neurocognitive performance or symptoms were observed, indicating recovery regardless of MC phase. |
| Roby et al. (2023) | Prospective cohort study | | 512 adolescent females (i.e., concussed), (age 15.2 ± 1.4 years) | Menstruation (Days 0-4), follicular (Days 5-14), early (Days 15-21), and late luteal phase (Days 22-28) | Not measured | Self-reported menstrual cycle phases | Sports-related concussion and history of previous concussion (Injury cause not specified) | Post-Concussion Symptom Inventory (PCSI) | Initial visit (12.5 ± 7.2 days postconcussion), Follow-up visit (3–4 months postconcussion) | MC phase at the time of injury was associated with symptom endorsement at follow up, with more symptoms reported for injuries during menstruation and early LP. |
| Wunderle et al. (2014) | Nested cohort study | | 144 females i.e., concussed), 1. SP users (age 26.5 ± 9.28 years, n = 35) 2. FP (age 32.6 ± 11.11, n = 72) 3. LP (age 30.6 ± 10.17, n = 37) | Follicular and luteal phase (no day range specified) | Progesterone levels assessed from blood samples (serum) | Self-reported menstrual cycle phases | Severe (i.e., motor vehicle accident with ejection or pedestrian struck by vehicle) and not severe (i.e., all other mechanisms) | Rivermead Post Concussion Questionnaire (RPCQ) and EuroQoL/EQ5D | 1 month after injury | Females injured during the LP had significantly worse outcomes at 1 month compared to those injured during the FP. There was no clear difference in outcomes between SP users and FP groups. |

MC, menstrual cycle; LP, luteal phase; FP, follicular phase; HC, hormonal contraceptives; OCP, oral contraceptive pills; SP users, synthetic progestin users (including OCP, IUD, Depo-Provera, Levonorgestrel Implants); SRC, sports-related concussion; N/A, not available.
